# Ischemic-Free Liver Transplantation Reduces the Recurrence of Hepatocellular Carcinoma After Liver Transplantation

**DOI:** 10.3389/fonc.2021.773535

**Published:** 2021-12-13

**Authors:** Yunhua Tang, Tielong Wang, Weiqiang Ju, Fangcong Li, Qi Zhang, Zhitao Chen, Jinlong Gong, Qiang Zhao, Dongping Wang, Maogen Chen, Zhiyong Guo, Xiaoshun He

**Affiliations:** ^1^ Organ Transplant Center, The First Affiliated Hospital, Sun Yat-sen University, Guangzhou, China; ^2^ Guangdong Provincial Key Laboratory of Organ Donation and Transplant Immunology, Guangzhou, China; ^3^ Guangdong Provincial International Cooperation Base of Science and Technology (Organ Transplantation), Guangzhou, China

**Keywords:** ischemia reperfusion injury, hepatocellular carcinoma, ischemic-free liver transplantation, prognosis, propensity-matched analysis

## Abstract

Ischemia reperfusion injury (IRI) is an adverse factor for hepatocellular carcinoma (HCC) recurrence after liver transplantation. Ischemic-free liver transplantation (IFLT) is a novel transplant procedure that can largely reduce or even prevent IRI, but the clinical relevance of IFLT and the recurrence of HCC after liver transplantation are still unknown. This retrospective study compared survival outcomes, HCC recurrence, perioperative data and IRI severity following liver transplantation (LT). 30 patients received IFLT and 196 patients received conventional liver transplantation (CLT) were chosen for the entire cohort between June 2017 and August 2020. A 1:3 propensity score matching was performed, 30 IFLT recipients and 85 matched CLT patients were enrolled in propensity-matched cohorts. An univariate and multivariate Cox regression analysis was performed, and showed surgical procedure (CLT *vs* IFLT) was an independent prognostic factor (HR 3.728, 95% CI 1.172-11.861, *P*=0.026) for recurrence free survival (RFS) in HCC patients following liver transplantation. In the Kaplan–Meier analysis, the RFS rates at 1 and 3 years after LT in recipients with HCC in the IFLT group were significantly higher than those in the CLT group both in the entire cohort and propensity-matched cohort (*P*=0.006 and *P*=0.048, respectively). In addition, patients in the IFLT group had a lower serum lactate level, lower serum ALT level and serum AST level on postoperative Day 1. LT recipients with HCC in the IFLT group had a lower incidence of early allograft dysfunction than LT recipients with HCC in the CLT group. Histological analysis showed no obvious hepatocyte necrosis or apoptosis in IFLT group. In conclusion, IFLT can significantly reduce IRI damage and has the potential to be a useful strategy to reduce HCC recurrence after liver transplantation.

## Introduction

Hepatocellular carcinoma (HCC) is the fifth most common cancer and the third cause of cancer-related mortality worldwide (1). Liver transplantation (LT) offers the most effective treatment for selected patients with HCC compared to liver resection or local ablation (2). However, the high incidence of postoperative recurrence has become a major concern and remains the main limitation of long-term outcomes of liver transplantation (3). Risk factors for HCC recurrence have been extensively investigated and are related to tumor size and number, microvascular invasion and poorly differentiated tumor grade (4, 5). In addition to tumor biology, increasing animal studies and clinical evidence suggest that ischemia reperfusion injury (IRI) promotes the recurrence of HCC after liver transplantation (6–8).

Liver grafts will inevitably be subject to varying degrees of IRI when organ procurement occurs after rapid cold flush, subsequent cold preservation and warm reperfusion after implantation into the recipient. Several studies have shown that liver IRI results in microvascular dysfunction, immune cell recruitment to liver grafts, and the release of pro-inflammatory and pro-proliferation mediators, facilitating the growth of circulating liver cancer cells in the injured liver (9, 10). For decades, great efforts have been made to reverse IRI and reduce the recurrence of HCC after liver transplantation, including ischemia preconditioning, immunological therapy and gene therapy (6, 11–13). However, few studies could be translated to the clinic. Obviously, none of the reported methods could effectively prevent IRI, which is an inevitable consequence due to cold preservation during liver transplantation. Ischemia-free liver transplantation (IFLT) is a novel transplant procedure that is able to procure, preserve and implant liver grafts without stopping the blood and oxygen supply for liver grafts. It has been well established that IRI was largely alleviated and even entirely prevented in IFLT in our previous studies (14, 15). However, to date, no reports have examined the clinical relevance of IFLT and the recurrence of HCC after liver transplantation.

In this study, we aimed to compare the transplant outcomes and graft IRI severity in recipients with HCC between IFLT and conventional liver transplantation (CLT), further assessing the impact of IFLT on the risk of HCC recurrence after liver transplantation.

## Patients and Methods

### Study Population

This was a retrospective cohort design study. We included adult (18 years of age and older) patients diagnosed with HCC preoperatively who underwent CLT or IFLT at The First Affiliated Hospital of Sun Yat-sen University, Guangzhou, China, between June 2017 and August 2020. Split LT, liver-kidney combined transplantation or multivisceral transplantation were excluded, and patients who died within 30 days of LT were also excluded. Because patients who underwent IFLT only received donors from donation after brain death (DBD), donation after circulatory death (DCD) or living donors were excluded from the current study. Thus, patients identified as having HCC were chosen for the study population (n=226); 30 of 226 patients received IFLT, and the remaining 196 patients received CLT. All 226 donors were enrolled in a voluntary organ donation program for deceased Chinese citizens, and informed consent was obtained from relatives of the donors. No organ donations were from executed prisoners. This study was approved by the Institutional Review Board of the First Affiliated Hospital of Sun Yat-sen University.

The donor variables were age, BMI, donor serum creatinine, donor total bilirubin, donor serum sodium, and cold ischemia time. The recipient variables were age at transplant, BMI, preoperative laboratory MELD score, and positive hepatitis B surface antigen. Tumor parameters were pretransplant AFP, most radiologic tumor diameter, number of lesions (1,2, 3+), Milan criteria, liver resection history, neoadjuvant therapy (RFA or TACE), tumor differentiation (well, moderate, poor), and microvascular invasion. Intraoperative and posttransplantation data included operation duration, anhepatic phase, total blood loss, blood transfusion, ICU stay, hospital stay, early allograft dysfunction (EAD), serum INR, lactate, ALT, AST and creatinine level on the first day posttransplantation, recurrence, date of recurrence, and site of recurrence.

To evaluate whether the outcomes of HCC recipients who underwent IFLT or CLT were different, propensity-matched analyses were performed. IFLT recipients were matched 1:3 with patients who had undergone CLT during the same time period utilizing a propensity match score, matching for the following variables: pretransplant tumor characteristics (serum AFP, the most radiologic tumor diameter, multiple/single, portal vein tumor thrombosis, liver resection history, RFA/TACE neoadjuvant therapy) and explant tumor characteristics (differentiation and microvascular invasion). The caliper width was 0.2 standard deviations of the logit-transformed propensity score. The absolute standardized differences method was used to diagnose the balance after matching, and all were confirmed to be less than 0.25.

The primary endpoint of this study was tumor recurrence. Secondary endpoints included operation time, intensive care and hospital stay, EAD, serum ALT and AST levels on the first day posttransplantation, histological analysis of liver tissues before procurement, at the end of preservation and after revascularization, and overall survival. EAD was defined by the presence of one or more of the following after LT (16): bilirubin of ≥10 mg/dL on Day 7, international normalized ratio of ≥1.6 on Day 7, and alanine aminotransferase or aspartate aminotransferase >2000 IU/L within the first 7 days. To estimate overall survival, survival time was calculated from the date of LT to death or last known follow-up, and status was recorded at the last point of contact with the patient who died or lived. To estimate recurrence-free survival (RFS), patients with no evidence of recurrence were censored at the time, of last follow-up or death.

### CLT and IFLT Procedure

**Figure 1:**
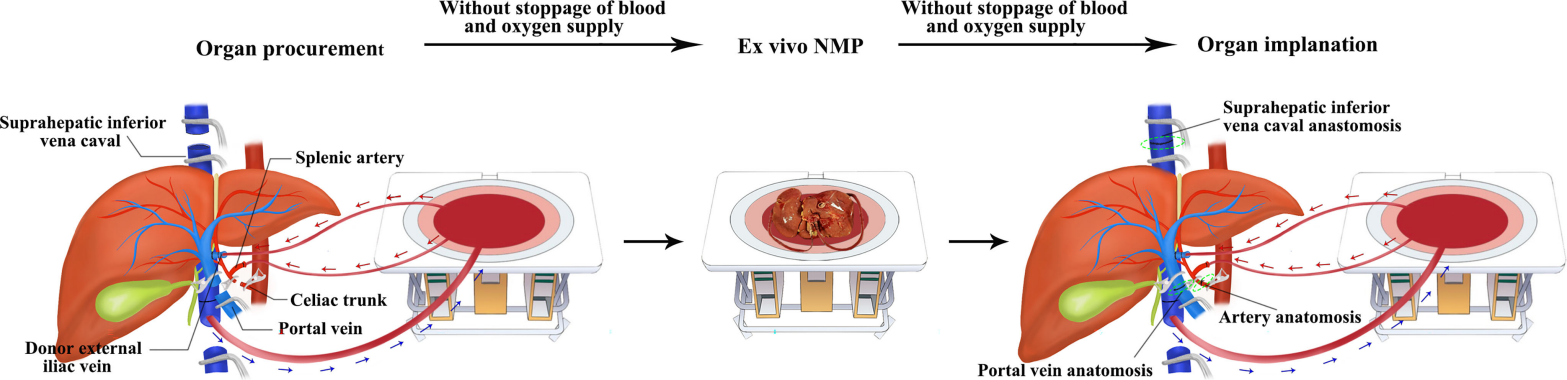
Graphic rendering of the ischemia-free liver transplantation procedure.

The CLT procedure included organ procurement after rapid cold flush, subsequent static cold storage (SCS) and back-table preparation, and then implantation. The surgical procedures of IFLT were as described in our previous study (14, 15). Briefly, after the liver was fully mobilized, a 4 cm-long segment of the external iliac vein from the blood donor was harvested and end-to-side anastomosed to the portal vein of liver donors, serving as an access point for portal vein cannulation while still permitting native blood flow through the portal vein. A 12 Fr cannula was inserted into the splenic artery (or gastroduodenal artery) without interruption of arterial supply to the liver from the celiac artery. A 32 Fr cannula was placed in the infrahepatic inferior vena cava for outflow. A straight 24 Fr cannula connected to the portal vein perfusion line of Liver Assist (Organ Assist, Groningen, The Netherlands) was inserted into the portal vein *via* the interposition vein. The arterial cannula was then connected to the hepatic artery (HA) perfusion line of Liver Assist. After the *in situ* normothermic mechanical perfusion (NMP) circuit for the livers was established, the livers were harvested and moved to the organ reservoir under continuous NMP. So that the graft did not suffer ischemia during procurement. Hereafter, the liver underwent ex situ NMP. The perfusate contained approximately 1.3 L cross-matched leucocyte-depleted washed red cells, 1.4 L Succinylated gelatinor, 30 mL 5% sodium bicarbonate, 0.5 g metronidazole, 37500 U heparin, 1.5 g cefoperazone sodium and sulbactam sodium, 30 mL 10% calcium gluconate, 3 mL 25% magnesium sulfate and 250 mL compound amino acid injection. In IFLT, no back-table preparation is needed, and the liver is implanted under continuous NMP. Briefly, the splenic artery (or gastroduodenal artery) and interposition vein and all vascular anastomoses (donor suprahepatic vena cava to recipient suprahepatic vena cava, donor portal vein to recipient portal vein, and donor celiac artery/common hepatic artery to recipient common hepatic artery) were performed without interruption of the blood supply to the graft under continuous NMP. After the native blood supply from the recipient’s portal vein and hepatic artery to the graft was re-established, the *in situ* NMP was stopped, and the cannulas were removed. The donor splenic artery (or gastroduodenal artery) was ligated closed, and the interposition vein was sutured closed. The donor infrahepatic vena cava and common bile duct were anastomosed to the recipient counterparts. Graphic rendering of the ischemia-free liver transplantation procedure is shown in **Figure 1**.

### Sample Collection

In *ex vivo* perfusion, samples from the perfusate were collected for analysis of the blood gas parameters (pO2, pCO2, PH and lactate). Perfusate samples were also collected and centrifuged, and the supernatant was stored at -80°C for liver function tests, such as aspartate aminotransferase (AST), alanine aminotransferase (ALT), and total bilirubin (Tbil), using standard biochemical methods. Bile production was collected every 60 minutes from the biliary draining tube for bile production calculation and detection of PH and HCO3^-^ of bile. Liver tissue biopsy was performed for histology studies before procurement, at the end of preservation and after revascularization, including hematoxylin and eosin (HE) staining for histological scoring and terminal deoxynucleotidyl transferase dUTP nick end labeling (TUNEL) assay for hepatocyte apoptosis.

### Transplantation Procedures and Postoperative Management

After CLT or IFLT, all patients were admitted to the intensive care unit. The immunosuppressive regimen was 20 mg basiliximab induction therapy administered during the operation and on postoperative Day 4. Both a calcineurin inhibitor and mycophenolate mofetil were administered for immunosuppressive maintenance therapy beginning on postoperative Day 4.

### Statistical Analysis

The donor and recipient characteristics were expressed as the means ± standard deviation (SD) for metric parameters and as percentages for nominal parameters. Continuous data were compared using t-tests, whereas categorical variables were compared using the chi-square test or Fisher’s exact test. We used Kaplan-Meier statistics and the log-rank test to analyze the recurrence-free survival (RFS) and overall survival (OS) rates of the patients both in the entire cohort and propensity-matched cohorts. To identify independent predictors of RFS in entire cohort, an univariate and multivariate Cox proportional hazard regression was conducted for all candidate predictors of RFS. Pretransplant serum AFP levels were categorized into two groups according to interquartile range 3 (IQR3). Variables with a *P* < 0.05 in univariate analysis were subjected to multivariate Cox proportional hazards regression *via* the forward stepwise method; the results were presented as hazard ratios (HRs) with 95% confidence intervals (CIs). The data analysis was performed using STATA 14.0 software (Stata Corp). *P <*0.05 was considered to be statistically significant.

## Results

### Recipient and Donor Clinical Characteristics Between the IFLT group and CLT Group

Patients identified as having HCC were chosen for the study population (n=226) between June 2017 and August 2020 in the entire cohort; 30 of 226 patients received IFLT, and the remaining 196 patients received CLT. To correct selection biases and confounding factors, propensity score matching was performed at a 1:3 ratio. After matching, 30 IFLT recipients and 85 matched CLT patients from our center were enrolled in propensity-matched cohorts during the same period. In the entire cohort, the duration of follow-up was 22.9 ± 11.2 months in the IFLT group and 22.6 ± 11.4 months in the CLT group with no significant differences, securing a minimal follow-up of 6 months in the two groups.

There were no significant differences in donor and recipient characteristics between the IFLT group and the CLT group both in the entire cohort and in the propensity-matched cohort, including donor characteristics (age, gender, donor BMI, donor serum creatinine, donor serum total bilirubin and donor serum sodium) and recipient characteristics (age, gender, BMI, laboratory MELD scores and positive hepatitis B surface antigen). The cold ischemia time in the CLT group was 6.7 ± 2.1 hours in the entire cohort and 6.8 ± 2.2 hours in the propensity-matched cohort. Donor and recipient characteristics were summarized in **Table 1**.

**Table 1:** Recipient and donor characteristics in IFLT and CLT groups before and after propensity score matching.

	Entire cohort			Propensity-matched cohort		
	IFLT group (n = 30)	CLT group (n = 196)	P	IFLT group (n = 30)	CLT group (n = 85)	P
**Donor Characteristics**						
Donor age (years)	41.4 ± 14.2	36.8 ± 14.9	0.117	41.4 ± 14.2	35.1 ± 14.3	0.053
Gender: male	66.7% (20/30)	74.0% (145/196)	0.401	66.7% (20/30	71.8% (61/85)	0.599
BMI, kg/m^2^	22.3 ± 2.3	23.3 ± 9.8	0.771	22.3 ± 2.3	22.2 ± 3.1	0.896
Donor serum creatinine (ummol/L)	87.3 ± 67.2	139.2 ± 155.6	0.074	87.3 ± 67.2	125.6 ± 107.7	0.071
Donor total bilirubin (umol/L)	27.7 ± 21.5	23.4 ± 17.6	0.250	27.7 ± 21.5	22.9 ± 14.5	0.180
Donor serum sodium (mmol/L)	147.6 ± 12.6	149.7 ± 16.7	0.508	147.6 ± 12.6	148.3 ± 20.8	0.870
Cold ischemia time (hours)	NA	6.7 ± 2.1	NA	NA	6.8 ± 2.2	NA
**Recipient Characteristics**						
Age at transplant (years)	54.2 ± 9.9	50.2 ± 73	0.088	54.2 ± 9.9	50.74 ± 9.9	0.106
Gender: male	96.7% (29/30)	92.9% (182/196)	0.435	96.7% (29/30)	90.6% (77/85)	0.287
BMI, kg/m^2^	23.4 ± 3.1	23.2 ± 3.2	0.793	23.4 ± 3.1	23.5 ± 3.5	0.822
Preoperative lab MELD score	15.4 ± 7.7	13.7 ± 8.1	0.279	15.4 ± 7.7	13 ± 7.8	0.174
Positive Hepatitis B surface antigen	90.0% (27/30)	86.7% (170/196)	0.618	90.0% (27/30)	85.9% (73/85)	0.565
Tumor parameter						
Pretransplant AFP (ug/l)	167.9 ± 428.5	12899.4 ± 729.2	**0.016**	167.9 ± 428.5	97.9 ± 342.8	0.371
Size biggest HCC lesion (mm)	43.47 ± 16.7	54.23 ± 41.74	0.129	43.47 ± 16.7	44.5 ± 33.8	0.751
Number of lesions						
single	46.7% (14/30)	39.3% (77/196)	0.443	46.7% (14/30)	42.4% (36/85)	0.682
Multiple	53.3% (16/30)	60.7% (119/196)		53.3% (16/30)	57.6% (49/85)	
Within Milan criteria	56.7% (17/30)	36.2% (71/196)	**0.032**	56.7% (17/30)	48.2% (41/85)	0.427
tumor differentiation						
Well	3.3% (1/30)	3.1% (6/196)	0.994	3.3% (1/30)	4.7% (4/85)	0.839
Moderate	73.3% (22/30)	73.0%%(143/196)		73.3% (22/30)	76.5% (65/85)	
Poor	23.3% (7/30)	24.0% (47/196)		23.3% (7/30)	18.8% (16/85)	
Microvascular invasion	16.7% (5/30)	33.7% (66/196)	**0.042**	16.7% (5/30)	18.8% (16/85)	0.793
Liver resection history	23.3% (7/30)	17.9% (35/196)	0.772	23.3% (7/30)	21.2% (18/85)	0.806
neoadjuvant therapy (RFA or TACE)	46.7% (14/30)	52.0% (102/196)	0.328	46.7% (14/30)	52.9% (45/85)	0.554
Duration of follow-up (days)	22.9 ± 11.2	22.6 ± 11.4	0.917	22.9 ± 11.2	24.8 ± 11.9	0.441

The bold emphasis of P value means that the value less than 0.05 has statistical significance.

### Comparison of Tumor Parameters Between the IFLT Group and CLT Group

In the entire cohort, the pretransplant AFP level was higher in the CLT group than in the IFLT group (*P*=0.016). The percentage of LT recipients within the Milan criteria and microvascular invasion was higher in the CLT group than in the IFLT group (*P*=0.032 and *P* = 0.042, respectively). There were no differences in the size largest HCC lesion, number of lesions, tumor differentiation, liver resection history and neoadjuvant therapy (RFA or TACE) history between the two groups. In the propensity-matched cohort, there were no differences in all tumor parameters between the two groups. Comparison of tumor parameters were summarized in **Table 1**.

### IFLT Provides a Larger Benefit for the Reduction in Post-LT HCC Recurrence Than CLT

To analysis whether IFLT has potential as an independent prognostic factor in HCC patients following liver transplantation, Cox regression analysis was performed to examine RFS in the entire cohort. Univariate analysis indicated that pretransplant AFP (≥ 300 ug/l *vs.* < 300 ug/l)(HR 3.830, 95% CI 2.447-5.997, *P <*0.001), biggest HCC diameter (≥ 5cm *vs.* < 5cm) (HR 1.753, 95% CI 1.119-2.746, *P* =0.014), poor tumor differentiation (HR 2.738, 95% CI 1.226-6.112, *P* = 0.014), microvascular invasion (HR 3.453, 95% CI 2.213-5.388, *P <*0.001) and surgical procedure (CLT *vs* IFLT) (HR 4.371, 95% CI 1.371-13.864, *P* =0.012) were associated with RFS. Furthermore, multivariate analysis demonstrated that pretransplant AFP (≥ 300 ug/l *vs.* < 300 ug/l) (HR 2.262, 95% CI 1.597-4.318, *P <*0.001), microvascular invasion (HR 2.309, 95% CI 1.403-3.801, *P <*0.001) and surgical procedure (CLT *vs* IFLT) (HR 3.728, 95% CI 1.172-11.861, *P* =0.026) were independent prognostic factors for DFS in HCC patients following liver transplantation (**Table 2**).

**Table 2:** Univariate and multivariate analyses of risk factors for recurrence-free survival in the entire cohort.

	Univariate analysis			Multivariate analysis		
	HR	95% CI	P	HR	95% CI	P
Recipient Characteristics						
age at transplant (years)	0.980	0.959-1.001	0.062			
Gender: male *vs* female	1.030	0.448-2.371	0.944			
BMI, kg/m^2^	1.006	0.940-1.077	0.860			
Preoperative lab MELD score	1.008	0.979-1.037	0.599			
Positive Hepatitis B surface antigen	1.382	0.418-4.569	0.596			
Pretransplant AFP: ≥ 300 *vs.* < 300 ug/l	3.830	2.447-5.997	**<0.001**	2.626	1.597-4.318	**<0.001**
biggest HCC diameter: ≥ 5cm *vs.* < 5cm	1.753	1.119-2.746	**0.014**			
Tumor Number ( single *vs.* multiple)	1.009	0.643-1.582	0.969			
Tumor differentiation: Moderate *vs.* Well	1.702	0.801-3.614	0.167			
Tumor differentiation: Poor *vs.* Well	2.738	1.226-6.112	**0.014**			
Microvascular invasion	3.453	2.213-5.388	**<0.001**	2.309	1.403-3.801	**0.001**
Liver resection history	0.983	0.560-1.725	0.952			
Neoadjuvant therapy (RFA or TACE)	1.113	0.715-1.732	0.636			
Donor Characteristics						
Donor age (years)	1.002	0.987-1.017	0.824			
BMI, kg/m^2^	0.946	0.878-1.019	0.144			
Donor serum creatinine (ummol/L)	1.001	0.999-1.003	0.393			
Donor total bilirubin (umol/L)	0.994	0.980-1.008	0.382			
Donor serum sodium (mmol/L)	1.001	0.988-1.014	0.899			
surgical procedure: CLT *vs.* IFLT	4.371	1.371-13.864	**0.012**	3.728	1.172-11.861	**0.026**

The bold emphasis of P value means that the value less than 0.05 has statistical significance.

**Figure 2:**
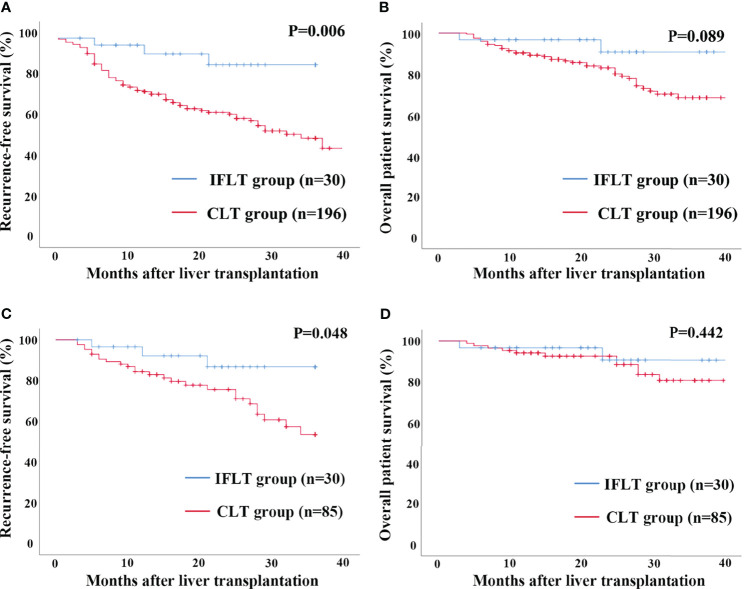
Recurrence-free survival and overall survival by Kaplan-Meier survival analysis for patients transplanted for HCC between the IFLT and CLT group for 3 years’ follow-up. **(A)** Recurrence-free survival in the entire cohort. **(B)** Overall survival in the entire cohort. **(C)** Recurrence-free survival in the propensity-matched cohort. **(D)** Overall survival in the propensity-matched cohort.

In the entire cohort, Kaplan–Meier analysis showed the RFS rates at 1 and 3 years after LT in recipients with HCC in the IFLT group were 92.2% and 86.7%, respectively, which were significantly higher than those (73.0% and 46.3%) in the CLT group (*P*=0.006, **Figure 2A**). The overall survival rates at 1 and 3 years after LT in recipients with HCC in the IFLT group were 96.7% and 90.6%, respectively, which tended to be higher than those (90.2% and 68.1%) in the CLT group, but with no significant differences (*P*=0.089, **Figure 2B**). In the propensity-matched cohort, the RFS rates at 1 and 3 years after LT in recipients with HCC in the IFLT group were 92.2% and 86.7%, respectively, which were significantly higher than those (88.1% and 53.6%, respectively) in the CLT group (*P*=0.048, **Figure 2C**). The overall survival rates at 1 and 3 years after LT in recipients with HCC in the IFLT group were 96.7% and 90.6%, respectively, which tended to be higher than those (94.1% and 70.6%) in the CLT group, but with no significant differences (*P*=0.442, **Figure 2D**). These results indicate that IFLT provides greater benefits than CLT in terms of the reduction in post-LT HCC recurrence.

### Comparison of Operative and Postoperative Outcomes Between the IFLT Group and CLT Group

In the entire cohort, LT recipients with HCC in the IFLT group had a shorter operation duration than LT recipients with HCC in the CLT group (6.3 ± 1.4 hours *vs.* 6.9 ± 1.5 hours, *P*=0.016). The anhepatic time was not different between the IFLT and CLT groups (52.2 ± 16.9 mins *vs.* 53.3 ± 14.8 mins, *P*=0.980). There were no differences in total blood loss or blood transfusion between the two groups. The ICU stay (52.4 ± 50.7 hours *vs.* 53.8 ± 50.2 hours, *P*=0.895) and hospital stay (23.8 ± 17.6 days *vs.* 25.8 ± 15.1 days, *P*=0.772) were similar between the two groups. LT recipients with HCC in the IFLT group had a lower incidence of EAD than LT recipients with HCC in the CLT group (3.3% *vs.* 29.6%, *P*=0.002). Patients in the IFLT group had a lower serum lactate level (1.9 ± 1.2 mmol/L *vs.* 2.7 ± 1.3 mmol/L, *P*=0.005), lower serum ALT level (198.8 ± 157.9 U/L *vs.* 633.8 ± 706.2 U/L, *P*=0.001) and serum AST level (437.1 ± 328.9 U/L *vs.* 1571.6 ± 1764.6 U/L, *P*=0.001) on postoperative Day 1. There were no differences in serum creatine levels or serum INR levels between the two groups on postoperative Day 1.

In the propensity-matched cohort, the indicators mentioned above had the same trend. Operative and postoperative outcomes are summarized in **Table 3**.

**Table 3:** Operative and postoperative outcomes in IFLT and CLT groups before and after propensity score matching.

	Entire cohort			Propensity-matched cohort		
	IFLT group (n = 30)	CLT group (n = 196)	P	IFLT group (n = 30)	CLT group (n = 85)	P
Operation duration (hours)	6.3 ± 1.4	6.9 ± 1.5	**0.016**	6.3 ± 1.4	7.0 ± 1.4	**0.017**
Anhepatic phase (mins)	52.2 ± 16.9	53.3 ± 14.8	0.980	52.2 ± 16.9	52.7 ± 13.2	0.086
Total blood loss (mL)	1726 ± 830	1626 ± 1649	0.775	1726 ± 830	1418 ± 1069	0.259
Blood transfusion (ml)	960 ± 860	940 ± 960	0.908	960 ± 860	820 ± 780	0.395
ICU stay (h)	52.4 ± 50.7	53.8 ± 50.2	0.895	52.4 ± 50.7	55.1 ± 47.2	0.798
Hospital stay (day)	23.8 ± 17.6	25.8 ± 15.1	0.772	23.8 ± 17.6	24.6 ± 14.1	0.816
EAD	3.3% (1/30)	29.6%(58/196)	**0.002**	3.3% (1/30)	29.4%(25/85)	**0.003**
INR day 1	1.5 ± 0.4	1.4 ± 0.3	0.179	1.5 ± 0.4	1.5 ± 0.3	0.575
Serum lactate day 1	1.9 ± 1.2	2.7 ± 1.3	**0.005**	1.9 ± 1.2	2.6 ± 1.3	**0.021**
ALT day 1, U/L	198.8 ± 157.9	633.8 ± 706.2	**0.001**	198.8 ± 157.9	617.6 ± 819.5	**0.007**
AST day 1, U/L	437.1 ± 328.9	1571.6 ± 1764.6	**0.001**	437.1 ± 328.9	1393 ± 1610	**0.002**
Creatinine day 1, mmol/L	95.7 ± 43.2	84.8 ± 35.3	0.129	95.7 ± 43.2	84.3 ± 37.3	0.186

The bold emphasis of P value means that the value less than 0.05 has statistical significance.

### IFLT Is Feasible, Safe and Effective, and Can Largely Reduce IRI in LT Recipients With HCC

**Figure 3:**
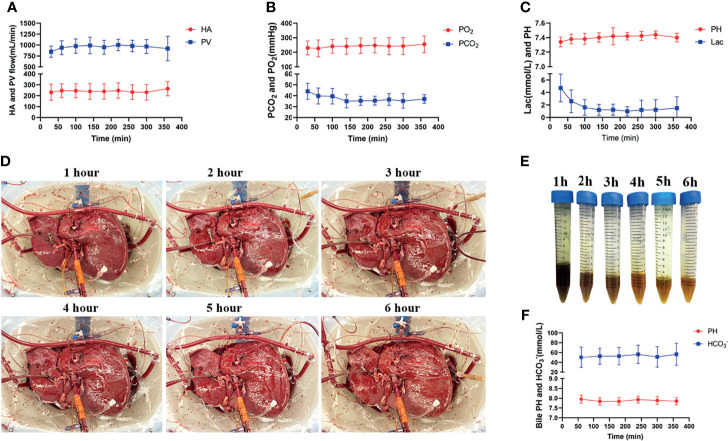
Normothermic machine perfusion. **(A)** The arterial and portal venous flow rates; **(B)** The O2 and CO2 tension in the perfusate. **(C)** pH values and lactate levels in the perfusate. **(D)** The grafts presented a vivid appearance during ex vivo perfusion; **(E)** The produced bile during ex vivo perfusion; **(F)** PH value and bicarbonate levels of the produced bile.

The NMP device provided adequate O2 and extraction of CO2 of the perfusion fluid with stable pressure and flow of both the portal vein and hepatic artery throughout the whole IFLT procedure (**Figures 3A, B**). The lactate levels in the perfusate dropped quickly from 4.73 ± 2.22 mmol/L to normal, and the pH value in the perfusate was within the normal physiological range, reflecting active metabolism by the liver grafts (**Figure 3C**). The liver grafts presented a vivid appearance during procurement, ex vivo NMP and implantation (**Figure 3D**). Continuous bile production with a high sodium bicarbonate and pH level indicated good quality of the bile (**Figures 3E, F**). Altogether, these results indicate the effectiveness of IFLT and suggest excellent organ viability.

**Figure 4:**
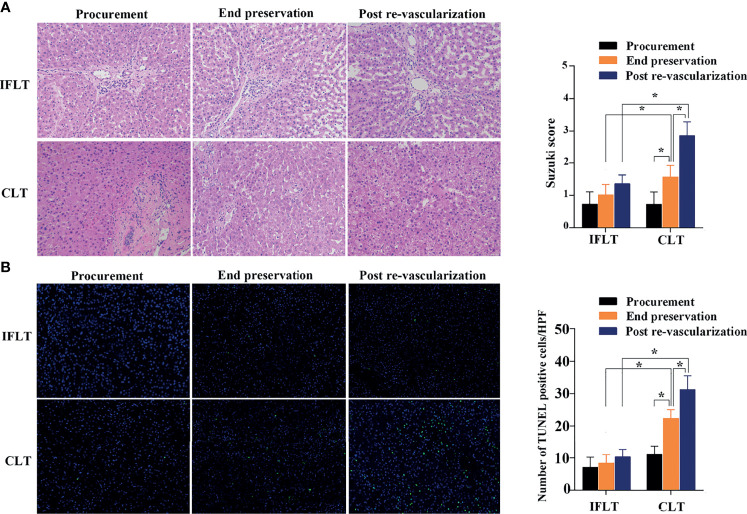
Histological analysis of liver tissues. **(A)** Hematoxylin and eosin (HE) of donor liver tissue biopsies before procurement, at the end of preservation and post-reperfusion in the IFLT and CLT. **(B)** The TdT-mediated dUTP nick end labelling (TUNEL) assay revealed that the number of apoptotic hepatocytes per high power field (HPF) before procurement, at the end of preservation and post-reperfusion in the IFLT and CLT. *P<0.001.

Hematoxylin and eosin (HE) staining evaluation of IFLT allograft biopsies showed stable and low Suzuki scores, whereas increased Suzuki scores were observed in the CLT allograft biopsies at the end of preservation graft and after revascularization (**Figure 4A**). The TUNEL assay showed no significant increase in apoptotic hepatocytes throughout the whole IFLT procedure in the IFLT group, while a significant increase in apoptotic hepatocytes was observed at the end of the preservation graft and after revascularization (**Figure 4B**). These results suggest that IFLT can largely reduce IRI in LT recipients with HCC.

## Discussion

IRI is an unavoidable adverse factor in liver transplantation. IRI can impair the function of transplanted organs, leading to EAD or even primary nonfunctioning (PNF), increasing the incidence of complications and mortality in recipients after surgery (17). In addition, IRI is associated with tumor recurrence and metastasis after liver transplantation. Much effort has been devoted to reducing the degree of IRI, including ischemia preconditioning and the use of protective gases, stem cells or gene therapy. However, few methods have been translated into clinical practice (18). Recently, great achievements have been made in *ex vivo* machine perfusion of organs. In particular, NMP could offer oxygen and nutrients and allow functional testing of liver grafts, which have been demonstrated to be obviously superior to static cold storage in minimizing IRI (19). In the current NMP setting, the organs still suffer cold storage injury and ischemia due to cold flush before procurement, back-table preparation on ice and normal saline flush after NMP. Research has shown that a short period of cold ischemia time still results in significant sinusoidal endothelial cell dysfunction and Kupffer cell activation in the liver (20). Therefore, we established IFLT without stopping the blood and oxygen supply and any cold preservation for the liver grafts during the whole transplant procedure. The protective effect of IFLT in reducing IRI on the donor liver was obvious. Peak transaminase levels within 1 week after LT correlated with both cold ischemia time and warm ischemia time, which can reflect the severity of IRI (21) and is regarded as a well-defined surrogate marker for long-term graft function and survival (21). In the current study, the peak value of transaminase on posttransplant Day 1 in the IFLT group was significantly lower than that in the CLT group. In addition, EAD suggests the initial poor function of liver grafts and represents the clinical phenotype of severe IRI after liver transplantation. Our results showed that LT recipients with HCC in the IFLT group had a lower incidence of EAD than LT recipients with HCC in the CLT group (3.3% *vs.* 29.4%, *P*=0.003), which was also much lower than the incidence of EAD of 20.0–30.0% reported by other centers in Western countries (16, 22, 23). Furthermore, histological analysis showed no significant increase in the Suzuki score or apoptotic hepatocytes during the whole transplant procedure in the IFLT group. In the IFLT group, the amount of blood loss and blood transfusion did not increase, and the postoperative ICU hospital stay and total hospital stay did not increase. These results suggested that IFLT did not increase the difficulty and complexity of the operation, nor did it increase the complications and hospitalization costs. Together, these findings indicate that IRI can be largely prevented in IFLT.

Clinically, previous studies have shown that posttransplant cancer recurrence and metastasis are significantly correlated with many factors, including tumor size and number (24), microvascular invasion (25), elevated AFP level (26, 27) and poorly differentiated tumor grade (4). In addition to liver tumor biology itself, increasing evidence supports the adverse effect of liver ischemia on the risk of liver cancer recurrence after liver transplantation. Nagai et al. (28) showed that prolonged cold and warm ischemia times of the liver graft are independent predictors of HCC recurrence one year after liver transplantation. Orci et al. (29) confirmed that LT recipients of organs from DCD donors with long warm ischemia times had higher HCC recurrence after liver transplantation. Several transplantation centers have reported that the rate of HCC recurrence was significantly higher following living donor liver transplantation than following deceased donor liver transplantation because the liver graft from a living donor is usually small for the recipient and more vulnerable to IRI (30, 31). On the other hand, several attempts targeting graft IRI effectively decreased the risk of early HCC recurrence after liver transplantation. Kornberg et al. (6) reported that treating liver graft IRI with prostaglandin E1 significantly increased the recurrence-free survival rates of recipients with HCC. A mouse model mimicking the recurrence of HCC after liver transplantation suggested that remote ischemic preconditioning offers protection against ischemia-mediated accelerated HCC recurrence (8). Matteo et al. (32) reported that the performance of hypothermic oxygenated liver perfusion before liver implantation appears advantageous to protect against HCC recurrence after liver transplantation, despite extended tumor criteria. In the current study, IRI was largely prevented. Here, we compared the difference in the postoperative recurrence rate between the IFLT and CLT groups. To correct selection biases and confounding factors, propensity score matching was performed. The tumor parameters, including tumor size, number, AFP level, tumor grade, microvascular infiltration and preoperative downstage treatment, were consistent between the two groups in our study. Both groups of patients received DBD liver donation without warm ischemia time, and IRI can be largely reduced even completely prevented by performing IFLT. An univariate and multivariate Cox regression analysis was performed in our study, and showed surgical procedure (CLT *vs* IFLT) was an independent prognostic factor for RFS in HCC patients following liver transplantation. In the Kaplan–Meier analysis, the recurrence free survival rates at 1 and 3 years after LT in recipients with HCC in the IFLT group were significantly higher than those in the CLT group both in the entire cohort and propensity-matched cohort (P=0.006 and P=0.048, respectively). These results together indicated that IFLT provides greater benefit than CLT in terms of the reduction in post-LT HCC recurrence, which supports that IRI had an impact on tumor recurrence after liver transplantation and that preventing or reducing IRI can reduce the HCC recurrence rate after liver transplantation.

The significance of IFLT in liver transplantation for HCC may not only improve the prognosis but also may expand the donor pool. Organ shortages are a problem to be solved worldwide, and patients with HCC who exceed the Milan or UCSF standard rarely have the opportunity to obtain a suitable liver. Marginal organs have greater vulnerability to IRI, such as older donors and fatty livers (33). Because China still in its early stages in developing an organ donation system based on deceased citizens, most donations originate from primary care hospitals, because of the lack of both basic medical equipment and doctors experienced with managing donors, these donors often suffer from multiple risk factors such as unstable blood circulation, hypoproteinemia, infection, and electrolyte disturbance. To be honest, we are relatively cautious about donor selection. Therefore, our initial experience is often based with a very young donors, which is a limitation of the study. However, IFLT can protect these marginal donor livers from IRI damage. Our previous studies reported the successful use of marginal donor livers (such as with hyperbilirubinemia or 85–95% macrovesicular steatosis) in LT recipients using IFLT (14, 34). Therefore, we recommend increasing the utilization of extended donor criteria or marginal donor liver grafts in recipients with HCC by performing IFLT in the future due to the benefit of IFLT-mediated reduction in IRI and post-LT HCC recurrence.

Our research has several limitations. First, IFLT is only carried out in a single center, and the overall sample size is insufficient. In the future, we will promote IFLT technology in multiple centers to increase the number of HCC patients to verify the significance of IFLT in reducing postoperative tumor recurrence. Second, there were not enough cases to perform stratified studies for different tumor stages. We will increase the number of HCC patients at different stages to explore the significance of IFLT in HCC patients within the standard or beyond the standard. Third, IFLT technology is currently limited to DBD donors, and we will explore the implementation of IFLT on DCD donors in the future. At that time, whether IFLT can reduce the recurrence rate of tumors after DCD donor liver transplantation still needs further research.

In conclusion, clinically, IRI increases the recurrence rate of HCC after liver transplantation. IFLT can significantly reduce IRI damage and has the potential to be a useful strategy to reduce HCC recurrence after liver transplantation.

## Data Availability Statement

The original contributions presented in the study are included in the article/supplementary materials, further inquiries can be directed to the corresponding author/s.

## Ethics Statement

All the procedures were performed in accordance with the ethical standards of the responsible committee on human experimentation (institutional and national) and with the Helsinki Declaration of 1964 and later versions. The study was approved by the Institutional Ethics Committee for Clinical Research and Animal Trials of the First Affiliated Hospital of Sun Yat-sen University and informed consent waiver was granted by the IEC given the retrospective, minimal risk nature of the study. No organs from executed prisoners were transplanted into any of the patients reported in this study.

## Author Contributions

YT and TW were responsible for writing the manuscript. FL, QiZ, ZC, and JG were responsible for data collection and statistics. WJ, QiaZ, DW, and MC were responsible for the revision of the manuscript. ZG and XH were responsible for the design of the project. All authors contributed to the article and approved the submitted version.

## Funding

Supported by grants as follows: the National Natural Science Foundation of China (81970564, 81471583, 81570587 and 81770410), Guangdong Basic and Applied Basic Research Foundation (2020A1515011557), the Key Clinical Specialty Construction Project of National Health and Family Planning Commission of the People’s Republic of China, the Guangdong Provincial Key Laboratory Construction Projection on Organ Donation and Transplant Immunology (2013A061401007, 2017B030314018), Guangdong Provincial international Cooperation Base of Science and Technology (Organ Transplantation) (2015B050501002), Guangdong Provincial Natural Science Funds for Major Basic Science Culture Project (2015A030308010), Guangdong Provincial Natural Science Funds for Distinguished Young Scholars (2015A030306025), Special support program for training high level talents in Guangdong Province (2015TQ01R168), Pearl River Nova Program of Guangzhou (201506010014), Science and Technology Program of Guangzhou (201704020150), Sun Yat-sen University Young Teacher Key Cultivate Project (17ykzd29) and “Elite program” specially supported by China organ transplantation development foundation.

## Conflict of Interest

The authors declare that the research was conducted in the absence of any commercial or financial relationships that could be construed as a potential conflict of interest.

## Publisher’s Note

All claims expressed in this article are solely those of the authors and do not necessarily represent those of their affiliated organizations, or those of the publisher, the editors and the reviewers. Any product that may be evaluated in this article, or claim that may be made by its manufacturer, is not guaranteed or endorsed by the publisher.

## Abbreviations

IRI, ischemia reperfusion injury; HCC, hepatocellular carcinoma; IFLT, ischemic-free liver transplantation; LT, liver transplantation; CLT, conventional liver transplantation; ALT, alanine aminotransferase; AST, aspartate aminotransferase; EAD, early allograft dysfunction; DBD, donation after brain death; DCD, donation after circulatory death; BMI, body mass index; MELD, Model for End-Stage Liver Disease; AFP, alpha-fetoprotein; RFA, radiofrequency ablation; TACE, transcatheter arterial chemoembolization; ICU, intensive care unit; SCS, static cold storage; HA, hepatic artery; NMP, normothermic machine perfusion; HE, hematoxylin and eosin; TUNEL, terminal deoxynucleotidyl transferase dUTP nick end labeling; SD, means ± standard deviation; RFS, recurrence-free survival; OS, overall survival; PNF, primary nonfunctioning; POD, post-operative day.
